# How and why do countries make Universal Health Care policies? Interplay of country and global factors

**DOI:** 10.7189/jogh.11.16003

**Published:** 2021-11-20

**Authors:** Chris Atim, Augustina Koduah, Soonman Kwon

**Affiliations:** 1 Results for Development, Accra, Ghana; 2 University of Ghana, Accra, Ghana; 3 Seoul National University, Seoul, South Korea

## Abstract

**Background:**

An examination of country policy making tends to reveal more complex processes that reflect domestic as well as external pressures and influences. The paper examines the interplay of external and internal, as well as other, factors in universal health care (UHC) decision-making for a select number of countries spanning the income range from low to high income.

**Methods:**

After developing a conceptual framework to help identify variables to explore in answering our study questions, we reviewed literature on health policies and policy making, especially around the time of the adoption of relevant policies for a number of UHC reform countries, followed by a narrative review of countries for more in-depth study. For more quantitative data, we consulted databases maintained by international institutions.

**Results:**

We found that, for low-income countries (LICs)/lower-middle-income countries (LMICs), the external environment helps set the policy agenda that drives national priorities and resource allocation decisions, while national actors take the actual decisions consistent with the interests of their constituencies and their goals. The upper-middle-income countries (UMICs) and high-income countries (HICs) in the study were less influenced by externally driven agendas and more by their own internal dynamics. For LICs/LMICs, a country’s income level as well as growth record did not appear to play any overt role at the start of the reform, whereas the UMIC/HIC countries were generally at a higher economic stage with steady growth when they initiated the reforms. The use of technical analysis and evidence to guide the UHC reform decisions was much more pronounced in the UMICs/HIC. The findings on alignment of the UHC program to national health priorities were more mixed. On sustainability, the UMICs/HIC were much more likely than LICs/LMICs to phase in their reforms, whether in terms of the geographical extension of coverage, the population groups to be covered or the expansion of the benefit package in the course of time.

**Conclusions:**

The near-systematic use of scientific evidence by the UMICs/HIC to inform decisions on the path to UHC in contrast to the LICs/LMICs leads to the conclusion that some LICs/LMICs may have made less than optimal resource allocation decisions based on scanty evidence and factors not conducive to sustainability of their UHC efforts.

It is not simply a coincidence that the global movement to encourage countries to commit to achieving universal health care (UHC) is happening at the same time as individual countries at different income levels, including low income ones, are putting forward policies and programs to move towards this goal. There does not appear to be much debate that such international declarations and agendas have significantly influenced the widespread proliferation of country-level UHC programs that we witness around the world today, particularly in low-income and lower-middle-income countries (LICs/LMICs) [1-3]. UHC policies or programs in this paper include explicit universal access programs and policies introduced by governments in health as well as policies and programs that aim to abolish, or significantly reduce the impact of, user fees in health, and/or extend or expand population access to health services, with the overall or eventual end goal of reaching the whole population.

The influence of international declarations or agendas may take the form of external pressures, international covenants and commitments and incentives such as the ready availability of technical and financial assistance to design and implement such policies. Some authors have also argued that the health policies of emerging market countries such as Brazil, Russia, India and China are also significantly affected by the agendas and policies set by international institutions and global commitments [4,5].

Do uncritical assumptions about the role of international agendas in country policy making and the institutions that promote them run the risk of ignoring the agency of countries themselves and the myriad circumstances that enter into their policy making? Would a closer examination of the actual decision-making processes reveal a more complex and dynamic picture, where, for instance, this global movement itself is largely a reflection and outcome of the interplay of country-level processes and initiatives and the countries’ interactions with the international community?

After all, it is also plausible that countries have had their own past experiences that possibly made them eager or predisposed to embrace new directions in health financing policy, and are riding on this favorable international context where a global movement is championing those new directions. Such past country-level experiences would include the consequences of structural adjustment programs and regressive user fee policies, and pressures from their own constituencies seeking the abolition of those unpopular user fees, or in the case of regimes that did not necessarily face electoral pressures, they may have nevertheless sought to implement popular social policies as part of their search for legitimacy.

We distinguish here between ‘developmentalist’ authoritarian regimes and other types of authoritarian regimes or dictatorships; the latter may hold on to power mainly by force and not appear to be bothered about seeking additional forms of legitimacy through rapid economic development and improvement of living standards, as is typical with the ‘developmentalist’ types discussed here [6]. While this paper deals only with the former type, we recognize that the latter type also exists and may have historically been more prevalent in Africa and other parts of the world.

The argument about country agency has particular force when applied to upper-middle-income countries (UMIC), which have tended to adopt UHC policies a lot earlier than the recent wave of ‘UHC-convertees.’ In those cases, and irrespective of prevailing international agendas or pressures, UHC policies “are often adopted in conjunction with a major social, economic, or political change. For example, UHC became a national priority following a period of financial crisis in Indonesia, Thailand, and Turkey; at the time of re-democratisation in Brazil” [7].

The main objective of the analysis is to try to understand factors that propelled, motivated and/or enabled some selected countries that have embarked on the journey towards UHC to make those decisions, including the interplay of external vs internal factors.

More specifically, this paper seeks to answer the following questions:

- What driving forces and factors determine/influence policy decisions on UHC?
  - What constituencies – internal and external – played a role in issue framing and decision-making processes, and which are the key or decisive ones?
  - What were enabling and facilitative factors versus actual country-driven solutions/approaches?
- What were the general trends and projections regarding income growth per capita at the time of the reform and how did such factors influence or impact the policy design, its implementation and declared goals?
- What was the role of evidence or data in informing the decision(s), if any?
- How was the UHC policy aligned with the country’s health priorities (as measured by the key health indicators that needed to be addressed)?
- How optimal were the investment and policy decisions around UHC when they were made? E.g. in addition to evidence that the decisions made or policies adopted were sustainable and aligned with national priorities of the time, were approaches such as piloting, sequencing or phasing considered at all?

An important dimension is also whether technical capacities were adequately assessed and if a program of capacity building was built into the process. The paper does not delve deeply into this beyond asking whether the process was evidence or data-driven.

## METHODOLOGY

We did a narrative review of a selection of countries representing the spectrum of LIC, LMIC, UMIC, and one high-income country (HIC), from across Africa, Asia and Latin America, the primary criteria for inclusion being that the country is perceived in the literature and UHC debates as having made some progress towards UHC in that region, or having some interesting or innovative features that offer lessons for others. Of the countries selected, there are more from Africa representing both the LIC and LMIC categories, which is particularly important for the study theme as the African countries have a greater degree of dependence and/or interactions with global institutions and development partners than those in other regions of the world.

Because of these inclusion criteria, the findings of this study are not generalizable to other countries and contexts, but other countries may draw lessons from the study ones.

**Figure 1:**
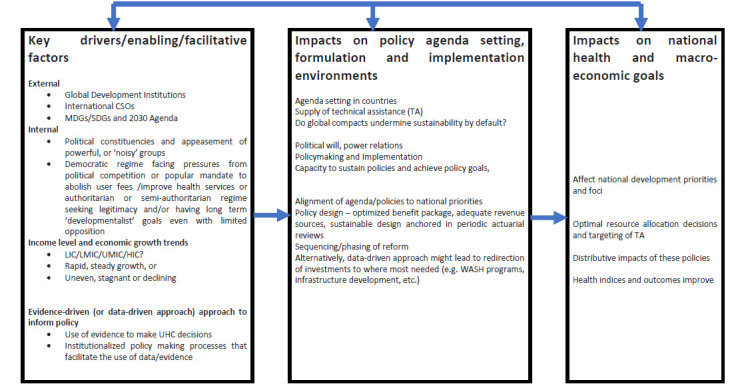
Conceptual framework for analysis.

A mixed methods approach is employed for the analysis, using quantitative and qualitative data and evidence. An analytical framework was first developed to frame the analysis and presentation of the findings, as shown in **Figure 1**. We then did a more in-depth examination of the factors that appeared to have played significant roles in the UHC decision-making processes of the selected countries.

It is also important to clarify that the study concerns principally the circumstances at the time the respective countries undertook their first major UHC reform, however where a country undertook UHC reforms over a period of time or in stages, an assessment of the overall process and the decision-making throughout the period are examined.

The data sources include published literature and other secondary sources pertaining to country policy making and other relevant aspects, while quantitative data are mainly from databases maintained by the Organisation for Economic Co-operation and Development (OECD), World Health Organization (WHO), World Bank Group (WB) and Institute for Health Metrics and Evaluation (IHME).

## RESULTS AND DISCUSSION

### Country UHC-related political context and period of reforms

The nature of the political regime of the country is usually an interplay of the constituencies that support and/or underpin the ruling government and the latter’s primary basis of legitimacy for its rule. The kind of political regime in each of the selected countries (**Table 1**) was ascertained by examining how political power is acquired and maintained in each country, whether there are different political parties with free competition of ideas and regular elections that the international community recognizes as being free and fair. A further differentiating factor in some cases is the ideology of the party holding power if it is a one-party state.

**Table 1:** Country UHC-related political context and period of reforms

Factors present at introduction of UHC program, unless otherwise stated	Kind of political regime	Time period of reform(s) studied
**Ethiopia**	Authoritarian, perceived as minority-based	2010
**Ghana**	Multi-party democracy	2003 NHIS law, amended 2012
**Nigeria**	Multi-party democracy	2005
**Rwanda**	Authoritarian, perceived as minority-based	1999 pilots and 2008 compulsory health insurance for all
**Senegal**	Multi-party democracy	2012-2014
**Tanzania**	Dominant one party with limited competition	2000 NHI Act and 2001 CHF Act
**Korea**	Authoritarian (introduction of NHI); democratic /competitive parties (UHC)	1989
**Vietnam**	Authoritarian, a single party socialist republic united under a communist government	1992, 2009
**Thailand**	Multi-party democracy	1997, 2002-2012
**Philippines**	Multi-party democracy	1995, 2017
**Mexico [**8,9**]**	Multi-party democracy	2001-2003
**Argentina [**8,9**]**	Multi-party democracy	2004, 2007-2012
**Chile [**8,9**]**	Multi-party democracy	2000-2005

The periods of the reforms undertaken by countries and examined in this study varied across countries, even over multiple years for many of them. Ethiopia introduced its health insurance strategy and a legal framework for it in 2010. Ghana passed its ambitious National Health Insurance Scheme (NHIS) law in 2003 and amended it in 2012 to include additional benefits while centralizing the institutional and governance framework.

Nigeria passed a national health insurance (NHI) scheme law in 2005, while Rwanda began a pilot program of community health insurance in 1999, followed in 2008 by a compulsory nationwide scheme with three regimes for different population groups. Senegal carried out pilot programs on community health insurance and enacted legislation to implement a nationwide, phased UHC program by 2014. Tanzania passed its National Health Insurance Act in 2000, targeting the formal sector, and the Community Health Fund (CHF) Act in 2001, meant to target the rest of the population.

The 1992 Vietnamese Constitution guaranteed health for all and the Government passed the Law on Social Health Insurance in 2009, creating a national Social Health Insurance program and combining disparate programs into a central policy. The Thai constitution acknowledged the right of every citizen to health care in 1997, while the Universal Coverage Scheme was implemented in 2002 with 70% population coverage and expanded to all by 2012. The Philippines embarked on an ambitious NHI program with the creation of PhilHealth in 1995 and the passage of the Universal Health Coverage bill 5784 by the House of Representatives in 2017.

The Argentine national constitution had guaranteed UHC since 1994. Plan Nacer, launched in 2003, achieved coverage of 2 million pregnant women and children by 2012. By 2015, Plan Nacer, which grew to include additional population groups through a new programme called Programa Sumar, covered 16 million children, adolescents, women and men [10]. In 2005, Chile introduced the Explicit Health Guarantees (GES) Plan, previously referred to as the Universal Access with Explicit Guarantees (AUGE) Plan. This Plan provides a series of legally enforceable guarantees, including a financial protection guarantee, timeliness guarantee and quality guarantee. Chile has since achieved 97% insurance coverage [8,9]. Mexico introduced the insurance program Seguro Popular in 2003, which specifically targets people without social security. Seguro Popular provides a guaranteed list of essential services for beneficiaries and covers approximately 45% of the population in Mexico as of 2012 [8].

### The interplay of external and internal drivers for UHC reforms

**Table 2** summarizes information showing that both external and internal factors appear to play key roles in the UHC decision-making processes for the LICs/LMICs; the external environment mainly sets the policy agenda that helps to drive national priorities and resource allocation decisions, while national actors take the actual decisions (and thus set the policy direction) consistent with the interests of their constituencies and their own political and socio-economic goals. The UMICs and HIC in the study were less influenced by externally driven agendas and tended to be driven more by the interests of their constituencies or their need for political survival (in the case of authoritarian regimes) through implementing popular social programs.

**Table 2:** Constituencies, facilitative environments and driving forces or factors

	Constituencies – internal	Constituencies – external	Enabling and facilitative environment
**Ethiopia**	General public, ruling party’s political and social base [11]	External support and TA played a key role in set up [11]	MDGs/SDGs, TA from development partners [12,13]
**Ghana**	General public, ruling political party [14,15]	External pressures had little to no role [14,15]	MDGs/SDGs [15-17]
**Nigeria**	Health Management Organizations (HMOs), private health care providers, federal employees [18]	External pressures had little to no role [18]	MDGs/SDGs, TA from development partners [16-18]
**Rwanda**	Ruling party’s political and social base [19]	External support and TA played a key role in set up [11,19]	MDGs/SDGs, TA from development partners [16,17]
**Senegal**	General public, ruling party’s political and social base [11]	Availability of external TA and funding was critical [11,13]	MDGs/SDGs, TA from development partners [13,16,17]
**Tanzania**	Ruling party’s political and social base [20]	Externally supported and driven pilot in 1990s led to later government design and roll-out [20]	MDGs/SDGs, TA from development partners [16,17]
**Korea**	Ruling party’s political and social base (UHC stage) [21-23]	External pressures had little to no role [21-23]	Rapid economic growth, market/economic reform [21-23]
**Vietnam**	Ruling party’s political and social base [24,25]	External pressures had little to no role [25]	Market/economic reform [25]
**Thailand**	General public, ruling party’s political and social base [24,26]	External pressures had little to no role [24]	Domestic expert groups [26]
**Philippines**	General public, ruling party’s political and social base [12]	External pressures had little to no role [12,27]	TA from developing partners [28]
**Mexico [**8,9**]**	General public, led by the Ministry of Health and its different branches [8]	External pressures had little to no role [8]	Health system reforms [8]
**Argentina [**8,9**]**	General public, ruling party’s political and social base [8]	External support and TA (WB) played an important role [8]	?
**Chile [**8,9**]**	General public, Presidency provided strong leadership [8]	External pressures had little to no role [8]	Long track record in public health, with system reforms in 1952 and 1981 [8]

It is also arguable that political will and commitment are important internal drivers in UHC decision-making processes in all countries. UHC is intrinsically political because it defines a set of policies based on social values, including the ideas of fairness and equity [24]. The study of the selected countries shows that political competition associated with democratization is a primary influence in the health financing reform process [29], as well as authoritarian regimes seeking legitimacy. Increased electoral competition can itself drive increased social provisioning as politicians appeal to excluded populations through social programs [30]. Additionally, UHC decisions form part of broader efforts of a government to foster legitimacy based on rapid and tangible socio-economic development [19].

The UHC decision-making processes of countries such as Ethiopia, Ghana, Rwanda and Chile benefited from high level political involvement of their leaders, who were aware of the benefits of working towards UHC and thus created a sense of urgency for such decisions among the technocrats and heads of relevant Ministries and agencies [9,19,24,30]. Chilean President Lagos’s unconditional support for UHC initiatives and his skillful maneuvering of the process neutralized powerful opposition that could have derailed the reforms [8]. Conversely, Nigerian political actors and policy makers appear to lack the level of political will and commitment required to make their decisions around UHC the priorities for all relevant actors [31], although other structural factors also play a part, such as an extreme form of federalism that gives much power to states to block, slow-walk or stall reforms [32].

Political support for UHC decisions creates an enabling environment for bureaucratic actors to frame and firm up UHC policy decisions and programs. With political support assured, the Ethiopian Ministry of Health’s bureaucrats spearheaded the decision-making processes with assistance from external consultants and development partners [30]. Political expediency can also enable trusted political associates, rather than bureaucrats, to take over final drafting of policy decisions, as was the case in Ghana [14].

Readily available technical assistance (TA) and financial support from global institutions and developed countries can influence UHC policy decisions and programs. Development partners from global institutions and developed countries can participate in other countries’ institutionalized policy making processes as policy agenda advocates and advisers, and by virtue of TA and funding, they help to frame the discourse to benefit global agendas [33]. The WB sponsored Argentina’s Program SUMAR to advance the country’s UHC strategy [34]. Development partners provided Nigeria, Rwanda, Senegal and Tanzania with technical and financial support [18].

Although development partners in Ghana supported a range of community-based health insurance (CBHI) schemes prior to the passage of the NHIS law in 2003, local actors ascribe no role at all to the development partners when describing the genesis of the Ghanaian NHIS design and policy decisions [14].

Rapid economic growth and governmental desires to foster legitimacy and maximize votes enabled and facilitated UHC policy decisions and programs in the Asian countries studied, rather than global agendas such as the Millennium Development Goals (MDG) and Sustainable Development Goals (SDG) [26] . In South Korea, a desire to maximize votes or political support played a crucial role in the rapid extension of NHI and achievement of UHC. A presidential election, in particular, triggered and expedited the extension of health insurance for universal coverage of the population. President Chun Doo-Whan and the presidential candidate of the ruling party, Roh Tae-Woo, were former military generals and wanted to obtain political support by proposing UHC. The 1987 presidential election was the first nation-wide election with the participation of the entire population in more than 25 years because previous presidents had been nominated through non-democratic indirect elections. This 1987 election pushed the ruling party to announce an expansion of social welfare programs as a major campaign agenda to maximize its voter support. In 1986, one year before the presidential election, the Government announced plans to extend NHI to the self-employed [23].

### Role of income level and economic growth record in UHC decision-making

**Figure 2:**
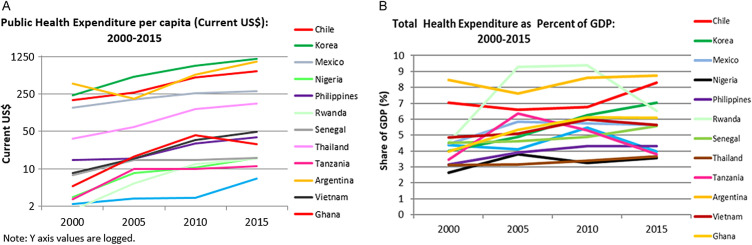
Health expenditure. **Panel A.** Public health expenditure per capita [35]. **Panel B.** Total health expenditure per capita [35].

The questions we tried to answer here include whether countries took their income level and economic growth prospects into account in making decisions about UHC - such as the size of the benefit package that was offered and the revenue sources and projections of the same – as well as whether, as a result of such considerations, phasing in of the policy and such issues played any part. These questions speak to the sustainability of the decisions made and, in particular, whether international agenda-setting and availability of TA and funding may have helped to skew resource allocation decisions away from optimality. We will discuss the income levels and general economic growth outlooks here (**Table 3**, **Figure 2**) and the broader sustainability issues below.

**Table 3:** Income level and growth trends at time of reforms

	Income level and growth trends at time of reform
**Ethiopia**	LIC, rapid and stellar economic growth (>6% per annum (p.a)) [35]
**Ghana**	LIC, steady but not stellar growth (<6% p.a.). Now LMIC [35]
**Nigeria**	LIC, mixed record of growth. Now LMIC [35]
**Rwanda [** 36 **]**	LIC, rapid and stellar growth [35]
**Senegal [** 36 **]**	LIC, steady but not stellar growth (<6% p.a.). Now LMIC [35]
**Tanzania [** 36 **]**	LIC, rapid and stellar growth [35]
**Korea [** 36 **]**	UMIC, rapid and slowing growth [21-23,35]
**Vietnam [** 36 **]**	LMIC, steady but not stellar growth (<6% p.a.) [35]
**Thailand [** 36 **]**	UMIC, after financial crisis and mixed record of growth [35]
**Philippines [** 36 **]**	LMIC, mixed record of growth [35]
**Mexico [**8,9**]**	UMIC, rapid and steady growth [8,35]
**Argentina [**8,9**]**	UMIC, rapid but uneven growth [8,35]
**Chile [**8,9**]**	UMIC, rapid and steady growth [8,35]

All the African countries in the study were LICs at the time of the inception of their UHC programs, including those which are now LMICs like Ghana and Nigeria. This is remarkable in itself because in previous historical periods, adopting a UHC program at that income level would have been considered unthinkable. The point is not to say that no LIC or LMIC should consider starting a UHC program; it is quite the contrary, as countries such as Rwanda and Sri Lanka have shown that a country at practically any income level can initiate a UHC program if it understands its socio-economic context and is willing to be guided by evidence and realism about what it can achieve within some given timeframes.

Much depends therefore on factors such as the content, ambition and timeline of the UHC program adopted. In that respect, several of the LIC/LMIC countries have proved quite cautious in managing the fiscal implications of their UHC policy. For instance, in defining the benefit package, Rwanda and Senegal both limited the package for some population groups due to budgetary considerations. With regards to resource mobilization for UHC, nearly all the LICs/LMICs fell short of identifying specific additional sources of revenue for their schemes, with Ghana, until recently, as a positive outlier for identifying a specific and highly-acclaimed innovative additional revenue source for UHC. Nigeria also more recently legislatively allocated one percent of the Federal Government’s Consolidated Revenue Fund for promoting coverage of a defined primary health care (PHC) package across the country.

In regard to phasing-in different population groups, Ethiopia, Nigeria, Senegal and Tanzania all limited access to their UHC programs; usually the formal sector receives the most extensive benefits and funding, while the informal sector is placed in a slower lane with CBHI schemes that offer varying but more limited benefits and lower population coverage (with the exception of Rwanda and possibly Ethiopia, whose CBHI programs appear to have advanced faster in covering large numbers of the informal sector). Ethiopia also appears to have been more deliberate in extending geographical coverage given fiscal and other capacity concerns. The program began with the implementation of CBHI schemes, which were piloted in 13 *woredas* (districts) based on a feasibility study. The program was then scaled up to other regions and *woredas.* Concurrently, with the establishment of the Ethiopian Health Insurance Agency (EHIA), which has federal and regional level offices, preparations are under way to set up the formal sector [37].

The Asian and Latin American countries in this study were generally at a more advanced income level and had more dynamic, steadily growing economies than the African ones when they embarked upon their UHC programs, and therefore faced more favorable conditions for the potential success of those programs. In a conventional sense, they had better justification for adopting such fiscally demanding initiatives, anticipating that future growth would be able to help contain or meet the rising health care costs of a benefit package commensurate with the expectations of their populations whose incomes and living standards are rising.

### Technical analysis and evidence for UHC decision-making processes

The use of evidence and data/information from other implementing countries, best practices, situational analysis from one’s context and treatment guidelines to inform country-specific UHC policy decisions and programs is critical and more likely to lead to successful design and implementation of a UHC program.

As the references for specific countries discussed in this section below show, not surprisingly, the MICs and HIC in the study made better use of technical analysis (a data-driven approach) in assessing whether to adopt the overall policy or not, but that approach was not systematically used by the LICs/LMICs. Ethiopia, Rwanda, and to some extent Senegal and Tanzania, made some use of evidence-driven policy making in a broad sense, either in the form of TA from partners to design the scheme or to design, implement and roll out prior pilot schemes. The use of more rigorous scientific evidence, such as clinical guidelines and treatment protocols, health technology assessments (HTA), calculations of expected costs per disability-adjusted life year (DALY) and actuarial assessments of the benefit package vis-à-vis the expected revenues, which was more common with the higher income countries in the study, was not a typical feature of the LIC/LMIC schemes.

In 2007–09, an Ethiopian team comprising Ministry of Health and external consultant staff conducted study tours to African and Asian countries. Lessons from the study tours informed the framing of Ethiopia’s CBHI design. Ethiopia's CBHI design was also strongly influenced and inspired by Rwanda's *Mutuelles de Santé* [30].

Ghana has one of Africa’s pioneering and most well-known NHI schemes, with the desire to cover the entire population within its ambit. However, the country’s UHC set up process was highly politically driven, to the practical exclusion and detriment of other, especially technical, considerations. While a large number of CBHI and mutual health organization (MHO) schemes already existed prior to the NHIS and could have served as de facto pilots for the NHIS design, the NHIS design team did not consciously seek to learn from and embed best practices and lessons from those smaller scale initiatives, apart from using an initially decentralized design (later abandoned) that built on the structure of pre-existing district-level mutual health insurance schemes. Similarly, although a near-contemporaneous actuarial study was conducted by the International Labour Organization (ILO) to assess the feasibility of the then emerging NHIS, there is no evidence that this work influenced the design, given obvious design flaws that were apparent even at birth. Incorporating lessons from existing CBHI/MHO schemes and/or findings from the ILO assessment could have led to a more sustainable and efficient scheme than the one that was eventually set up.

In Argentina, selected UHC services are backed by clinical guidelines and approved by the Ministry of Health and provincial health ministries. Through broad consensus, UHC policy decisions in Mexico were based on facts, including cost per DALY gained per beneficiary per year [24]. In Chile, the reform had a scientific basis that drew upon the perspective of the public and reflected its needs. Estimation of cost per case and total cost per condition were based on treatment protocols suggested by experts and scientific associations [8,9].

It is notable that the LIC/LMIC processes tend to proceed from a political decision made in advance (a phenomenon that is not unique to that set of countries), frequently coupled with granular specificity regarding politically palatable but technically challenging variables, such as the content of the benefit package and whether and what cost controls should be built in. Such prior decisions then frame the subsequent processes in a way that would not allow for ‘go’ or ‘no-go’ decisions to be made on those technical design variables that critically determine sustainability (eg, HTA can help decide what services should be included in the benefit package, but their usefulness is precluded if a prior high level political decision has been made to offer a specific benefit package).

### Alignment of UHC decisions with country health priorities

All countries would proclaim that their policies are of course in line with their national priorities. For this study, we took a rather narrow view of national health priorities, which measures, for an LIC/LMIC usually characterized by resource constraints more severe than other countries, whether the benefit package of the UHC program covers or focuses on the causes of underperformance on key health indicators, such as those pertaining to maternal, neo-natal, and child health. **Table 4** presents these findings for the countries studied.

**Table 4:** Alignment of UHC policy with health priorities

	UHC policy aligned with the country’s health priorities, as measured by health indicators
**Ethiopia**	Arguably yes [30,38]
**Ghana**	Arguably not (curative-focused health benefits plan not targeting key priorities) [14,33]
**Nigeria**	Arguably not at start, later modifications yes [18,39]
**Rwanda**	Arguably yes [36]
**Senegal**	Different benefit packages for population groups not specifically targeting PHC or similar priorities [11,13]
**Tanzania**	?
**Korea**	Arguably no (weak PHC) [22,23]
**Vietnam**	Arguably no [24,40]
**Thailand**	Arguably yes [26]
**Philippines**	Arguably no (limited financial protection in spite of 70%-80% population coverage by social health insurance) [27]
**Mexico [**8,9**]**	Yes [8]
**Argentina [**8,9**]**	Yes, Plan Nacer from 2003 based on PHC [8]
**Chile [**8,9**]**	Yes, but developed over time to reach that stage [8]

One sign that a country is not focused on targeting those causes is the situation where resources are sub-optimally allocated towards the higher levels of the health system, to the neglect or detriment of the PHC level, particularly preventive and promotive services. In most cases, therefore, focusing limited resources on health priorities translates into a situation where PHC services (including preventive and promotive activities) are fully covered under the UHC program.

Countries studied generally aligned UHC policy decisions on the health benefit package to national health priorities, although countries such as Ghana and Nigeria focused on curative rather than primary and preventive care [24,41]. The Nigerian national health policy, health financial policy, national health bill and national strategic health development plan (2010-2015) all focus on how to raise funds for health and move closer to UHC [42], but resource allocation is skewed in favor of secondary and tertiary care over PHC [41]. Ghana’s NHIS reimbursement policy excludes critical PHC functions, including preventive and promotive care. Indeed, national policy is to promote the Community Health Planning and Services (CHPS) as the base of the health system. While CHPS is connected to health centers in a satellite relationship and has a mission to reach all the population with PHC, including outreach and home visits, the NHIS would not pay for those services. This skewed the incentives towards making the CHPS units into curative care fixed stations where people go to seek care, rather than have community health officers go to the people in the communities [43]. However, Ethiopia’s 2002 health sector development plan and the subsequent UHC program focused on universal PHC [30].

By virtue of the greater amount of resources available to UMICs to meet the needs of their populations, as well as the much better use of evidence in making their resource allocation decisions, it would be expected that the UMICs in our study should all meet this assessment dimension of alignment of UHC programs with national priorities. Surprisingly however, three of the four Asian countries - including the only HIC in the study, Korea - have either weak or insufficient focus on PHC policies and practices, resulting in an increasing share of hospital-based care in the UHC program (**Table 4**). Thailand is the exception among them in strengthening PHC services in the UHC program.

The Latin American countries fare rather better on this dimension. Argentina’s constitution states that the public health system must provide free health services to all citizens who demand them, as needed and without exception. The private and social health insurance subsectors therefore have explicit health benefit plans, for which the Compulsory health plan establishes the general framework and indicates the services they must guarantee [8]. Chile and Mexico both cover PHC services in their programs (**Table 4**).

### Sustainability of UHC reform

Overall, it is arguable from this study that at least some of the LICs/LMICs, following the UHC 2030 agenda, have made sub-optimal resource allocation decisions and failed to properly assess the best use of scarce resources. When UHC policy decisions were made, important dimensions (other than alignment with the key health priorities discussed above), such as appropriate phasing, sequencing, national capacities and sustainability, were not always paramount in decision-making (**Table 5**). Sequencing of reforms “refers to the order in which either macroeconomic policy actions or specific reforms are introduced. Sequencing involves the order in which reforms are undertaken across sectors ... and the order in which reforms are undertaken within sectors ...” [45].

**Table 5:** Sustainability of design

Sustainability issues:	Design informed by costing/actuarial study	Phased approach (geographical, targeting, HBP size, other)	Sequencing of reforms
**Ethiopia**	Yes? [38]	Targeting of the informal sector phased [38]	Prior institutional reforms before UHC not clear [11,30]
**Ghana**	No [14]	None, practically [14]	None [14]
**Nigeria**	No [18,39]	Yes, federal employees first, other public sector and informal sector afterward [18,39]	Prior institutional reforms before UHC not clear [18]
**Rwanda**	Yes? [19,36]	Yes, formal sector first, informal sector phased(?) [19]	Yes, but institutional reforms lagging? [19]
**Senegal**	Yes, for pilots, not for expanded schemes [13]	Formal sector first, informal sector phased [11,13]	Sequencing not clear [13]
**Tanzania**	N.A.	Formal sector first, informal sector phased [20,44]	N.A.
**Korea**	Yes [21-23]	Yes, formal sector first, then all informal sector [21-23]	Prior institutional reforms (eg,, supply–side readiness, insurance agency institution building) before UHC [21,23]
**Vietnam**	No [24,25]	Yes, formal sector first, then informal sector phased [25,40]	None [5,24]
**Thailand**	Yes [26]	Yes, formal sector first, then all informal sector [24-26]	Prior institutional reforms (eg, supply–side readiness) before UHC [24,26]
**Philippines**	No [27]	Yes, formal sector first, then informal sector phased [12,27]	None [27]
**Mexico [**8,9**]**	Yes [8]	Yes, with segmentation of the population and different health benefit plans [8]	Prior institutional reforms before UHC [8]
**Argentina [**8,9**]**	?	Yes, Plan Nacer benefits phased in [8]	Yes [8]
**Chile [**8,9**]**	Yes [8]	Yes, different health benefit plans for different population groups [8]	Yes [8]

We found that the MICs, with notable exceptions, generally tended to pay greater attention to these factors in developing and implementing their UHC policies.

We found that the health system in Mexico is segmented, with the services received by the population dependent on individuals’ employment status and even ability to pay. The Mexican Ministry of Health began implementing the Coverage Expansion Plan (PAC) in 1996 with a strong emphasis on health promotion and prevention, directed especially at the rural poor. In 1997, this package was integrated into the conditional cash transfer program Progresa (Program for Education, Health and Nutrition), which was later renamed Oportunidades. The implementation of Seguro Popular was gradual; the program launched its operations in 2001 in five pilot states, with coverage of 59 500 low-income families. By December 2012, Seguro Popular had grown to cover approximately 52.9 million people, roughly 45% of Mexico’s population [8].

Similarly, in Argentina, implementation of the UHC package was phased. The implementation of the Plan Nacer (now Program SUMAR) was conducted in two phases. The first phase began in 2004 in the nine provinces of northern Argentina, and the plan was extended to the rest of the country in 2007. Plan Nacer began with a health benefit package that was tightly focused on just a few prioritized benefits and population groups, later taking a path of gradual expansion by adding “layers” of new population groups and services [8].

In South Korea, the government introduced mandatory SHI (also NHI) in 1977 and incrementally extended population coverage. In 1977, NHI was first applied to the poor and workers in big corporations with more than 500 employees. Two years later, public employees, schoolteachers, and workers in firms with more than 300 employees joined the NHI, followed by incremental extensions to employees of smaller business. In 1988, all rural self-employed individuals were covered by NHI, and a year later, universal coverage was achieved when the urban self-employed joined the NHI. In 2000, more than 300 health insurance funds were merged into a single payer NHI system, overcoming the challenges of a fragmented system [46].

The Ethiopian government targeted the informal sector and has been piloting and scaling up CBHI since 2010, and is establishing SHI for formal sector workers as a means of achieving UHC [30]. Additionally, the UHC scheme in Rwanda has evolved from a pure form of voluntary CBHI to one based on obligatory enrollment and subsidies from the formal sector, thus paving the way for a NHI model. Before the scheme became compulsory in 2006, it was already recognized as one of the rare successes of wide CBHI coverage in Sub-Saharan Africa [19]. The Nigerian government piloted CBHI on a small scale in Anambra, Lagos and Kwara States, but it was officially rolled out in Nigeria recently [42]. Senegal has also seen the development of numerous CBHIs. The first such scheme originated in 1989 in the Western part of the country near the capital of Dakar. While there has been substantial expansion of CBHIs throughout the country, there has yet to be any major efforts to organize or consolidate these schemes into a more national health insurance plan [29].

In Ghana, the NHIS was implemented without any phasing, whether of the benefit package or of the population groups covered. A key factor often cited by stakeholders in the country for the scheme’s chronic deficits and long delays in reimbursing providers, and even of the return of user fees for insured patients for supposedly insured services, is the ambitious design and implementation features without any form of phasing and irrespective of the economic and other capacities of the country [43].

## CONCLUSIONS

The study found that global policies and technical and financial support from donors and international institutions help to set the policy agenda and affect resource allocation decisions of LICs/LMICs, but have much less influence on the decision-making of UMICs and the HIC studied. However, all countries make policies based on their internal political and social dynamics, including the demands and aspirations of their own constituencies, the desire to maximize their share of the votes (where there is a competitive political environment) and the search for legitimacy and ‘developmentalist’ goals in an authoritarian regime context.

For the LICs/LMICs, their income level at inception did not appear to be a consideration in whether to start a UHC scheme or not. Countries at different levels of development have made decisions to start UHC programs, although the period when most of those countries in the study started their schemes coincided with positive, but not necessarily always stellar, economic growth and slowly rising income per capita. For the UMICs/HIC, the picture appears less ambiguous; they launched their UHC policies in a period of rapid economic and per capita income growth and the decisions taken (eg, on the benefit package) were affected by their economic circumstances and prospects.

The use of scientific evidence in the UHC decision-making process was much more pronounced in the UMICs/HIC, and less so in the LICs/LMICs. The former countries tended to use rigorous methods such as clinical guidelines and treatment protocols, HTA, actuarial assessments and costing and calculations of DALYs gained, while the latter ones tended to rely on TA from partners to design and implement their schemes, pilots in some cases, and study visits to learn from other country experiences.

From this analysis, the use of scientific evidence to inform decisions on the path to UHC presents perhaps the clearest contrast between the LICs/LMICs and the UMICs/HIC studied. Given how determinative the use of evidence can be for success (a sustainable design) or failure, this is an issue of concern about the manner in which UHC decisions are made and implemented in the LICs/LMICs. A further question that then suggests itself, but which we have not investigated, is whether this finding reflects a true lack of local capacities or whether, at least in some cases, there might have been substitution of external (or development partner) TA for the local capacities, which may have undermined sustainability.

Further, we also found that UHC policies tended broadly to align with country health priorities (as measured by adequate or complete coverage of PHC services, including prevention and promotion), with some notable exceptions that did not align neatly with the LIC/LMIC and UMIC/HIC divisions that we have seen throughout the rest of the study. This is a potential area for future research, as this result appears at face value to be rather counter-intuitive.

Most countries of different income levels tended to pay attention to the fiscal implications and hence financial sustainability of UHC design, by phasing in the implementation (including gradually increasing the population covered, the size of the benefit package, or the geographical coverage) and limiting the availability of the benefit package to different population groups (for LICs/LMICs in particular). The UMICs/HIC were almost all characterized by phased implementation over time until they reached high population coverage with more generous benefit packages. Arguably, this more careful approach to achieving UHC is one instructive manifestation of the above finding concerning the deliberate use of evidence and data to make UHC policy decisions by the UMICs/HIC, compared to the LICs/LMICs. Phasing in the reforms does however involve accepting a potential risk of fragmentation and social stratification.

## References

[1] American Public Health Association. Strengthening Health Systems in Developing Countries. 2008. Available: https://www.apha.org/policies-and-advocacy/public-health-policy-statements/policy-database/2014/07/23/09/09/strengthening-health-systems-in-developing-countries. Accessed: 17 June 2020.

[2] Bermeo S. Development, self-interest, and the countries left behind. 2018. Available: https://www.brookings.edu/blog/future-development/2018/02/07/development-self-interest-and-the-countries-left-behind/. Accessed: 17 June 2020.

[3] Patterson D London L International law, human rights and HIV/AIDS. Bull World Health Organ. 2002;80:964-9. 12571725 PMC2567707

[4] Sridhar D Gomez E Health Financing in Brazil, Russia and India: What Role Does the International Community Play? Health Policy Plan. 2011;26:12-24. 10.1093/heapol/czq016 20400535

[5] Huang Y International Institutions and China’s Health Policy. J Health Polit Policy Law. 2015;40:41-71. 10.1215/03616878-2854551 25480846

[6] Reinert E. Developmentalism. Working Papers in Technology Governance and Economic Dynamics. Tallinn, Estonia: Tallinn University of Technology Ragnar Nurkse Department of Innovation and Governance; 2010.

[7] Reich MR Harris J Ikegami N Maeda A Cashin C Araujo E Moving towards universal health coverage: lessons from 11 country studies. Lancet. 2016;387:811-6. 10.1016/S0140-6736(15)60002-2 26299185

[8] Giedion U, Tristao I, Escobar L, Bitrán R, Cañón O, Molins S, et al. Health Benefits Plans in Latin America: A Regional Comparison. Washington, DC: Inter-American Development Bank; 2014.

[9] Eduardo M, Solimano G. Towards Universal Health Coverage: the Chilean experience. World Health Report Background Paper No. 4. Geneva, Switzerland: World Health Organization; 2010.

[10] Glassman A, Temin M. Millions Saved: New Cases of Proven Success in Global. Washington, DC: Brookings Institution Press; 2016. Paying for Provincial Performance in Health: Argentina's Plan Nacer; 97-105.

[11] Zelelew H. Community Health Financing as a Pathway to Universal Health Coverage: Synthesis of Evidence from Ghana, Senegal, and Ethiopia. USAID Health Finance & Governance Project. Bethesda, MD: Abt Associates Inc.; 2015.

[12] Dayrit M, Lagrada L, Picazo O, Pons M, Villaverde M. (2018). The Philippines Health System Review. Vol. 8 No. 2. New Delhi: World Health Organization, Regional Office for South-East Asia; 2018.

[13] Tine J, Faye S, Nakhimovsky S, Hatt L. Universal Health Coverage Measurement in a Lower-Middle-Income Context: A Senegalese Case Study. Health Finance & Governance Project. Bethesda, MD: Abt Associates Inc.; 2014.

[14] Agyepong IA Adjei S Public social policy development and implementation: a case study of the Ghana National Health Insurance scheme. Health Policy Plan. 2008;23:150-60. 10.1093/heapol/czn002 18245803

[15] Darko T. How does government responsiveness come about? The politics of accountability in Ghana’s National Health Insurance Scheme. Brighton, UK: Institute of Development Studies; 2016.

[16] United Nations General Assembly. Resolution A/RES/55/2, United Nations Millennium Declaration. 2000.

[17] United Nations General Assembly. Transforming our world: the 2030 Agenda for Sustainable Development. Available: https://documents-dds-ny.un.org/doc/UNDOC/GEN/N15/291/89/PDF/N1529189.pdf?OpenElement. Accessed: 17 June 2020.

[18] Onoka CA Hanson K Hanefeld J Towards universal coverage: a policy analysis of the development of the National Health Insurance Scheme in Nigeria. Health Policy Plan. 2015;30:1105-17. 10.1093/heapol/czu116 25339634

[19] Chemouni B The political path to universal health coverage: Power, ideas and community-based health insurance in Rwanda. World Dev. 2018;106:87-98. 10.1016/j.worlddev.2018.01.023

[20] Todd G, Nswilla A, Kisanga O, Mamdani M. A case study of the Essential Health Benefit in Tanzania mainland. EQUINET Discussion Paper 109. Ifakara, Tanzania: Ifakara Health Institute; 2017.

[21] Na S, Kwon S. Building Systems for Universal Health Coverage in South Korea. Health, Nutrition and Population (HNP) Discussion Paper No. 98266. Washington, DC: World Bank Group; 2015.

[22] Kwon S. Advancing Universal Health Coverage: What Developing Countries Can Learn from the Korean Experience? Universal Health Coverage Studies Series. No. 33. Washington, DC: World Bank Group; 2018.

[23] Kwon S Thirty years of National Health Insurance in South Korea: lessons for achieving universal health care coverage. Health Policy Plan. 2009;24:63-71. 10.1093/heapol/czn037 19004861

[24] Rosenberg J, Madore A, Weintraub R. Concept Note: Implementing Universal Health Coverage: The Experience in Thailand, Ghana, Rwanda, and Vietnam. Boston, MA: Harvard Business Publishing; 2015.

[25] Kelsall T, Hart T, Laws E. Political settlements and pathways to universal health coverage. London: Overseas Development Institute; 2016.

[26] Tangcharoensathien V Patcharanarumol W Kulthanmanusorn A Saengruang N Kosiyaporn H The Political Economy of UHC Reform in Thailand: Lessons for Low- and Middle-Income Countries. Health Syst Reform. 2019;5:195-208. 10.1080/23288604.2019.1630595 31407962

[27] Picazo O, Ulep V, Pantig IM, Ho BL. A Critical Analysis of Purchasing of Health Services in the Philippines. A case study of PhilHealth. Discussion Paper No. 2015-54. Makati, Philippines: Philippine Institute for Development Studies; 2015.

[28] World Health Organization. UHC Act in the Philippines: a new dawn for health care. Available: https://www.who.int/philippines/news/feature-stories/detail/uhc-act-in-the-philippines-a-new-dawn-for-health-care.

[29] Grépin K Dionne KY Democratization and Universal Health Coverage: A Case Comparison of Ghana, Kenya, and Senegal. Glob Health Gov. 2013;6:1-27.

[30] Lavers T Towards Universal Health Coverage in Ethiopia’s ‘developmental state’? The political drivers of health insurance. Soc Sci Med. 2019;228:60-7. 10.1016/j.socscimed.2019.03.007 30884423

[31] Aregbeshola BS Enhancing Political Will for Universal Health Coverage in Nigeria. MEDICC Rev. 2017;19:42-6. 10.37757/MR2017.V19.N1.8 28225545

[32] Atim C, Bhatnagar A. Toward Synergy and Collaboration to Expand the Supply of and Strengthen Primary Health Care in Nigeria’s Federal Context, with Special Reference to Ondo State. UNICO Studies Series No. 3. Washington, DC: World Bank Group; 2013.

[33] Koduah A Agyepong IA van Dijk H The one with the purse makes policy’: Power, problem definition, framing and maternal health policies and programmes evolution in national level institutionalised policy making processes in Ghana. Soc Sci Med. 2016;167:79-87. 10.1016/j.socscimed.2016.08.051 27614028

[34] Rubinstein A Zerbino MC Cejas C López A Making Universal Health Care Effective in Argentina: A Blueprint for Reform. Health Syst Reform. 2018;4:203-13. 10.1080/23288604.2018.1477537 30067439

[35] World Bank Group. World Development Indicators. Available: https://databank.worldbank.org/reports.aspx?source=world-development-indicators. Accessed

[36] Ministry of Health. Republic of Rwanda. Fourth Health Sector Strategic Plan (HSSP 4): July 2018 – June 2024. Kigali, Rwanda: Ministry of Health; 2018.

[37] Alebachew A, Yusuf Y, Mann C, Berman P. Ethiopia’s Progress in Health Financing and the Contribution of the 1998 Health Care and Financing Strategy in Ethiopia. Resource Tracking and Management Project. Boston, MA and Addis Ababa, Ethiopia: Harvard T H Chan School of Public Health and Ethiopian Federal Ministry of Health; 2015.

[38] Federal Democratic Republic of Ethiopia Ministry of Health. Health Sector Transformation Plan (HSTP) 2015/16 - 2019/20. Addis Ababa, Ethiopia: Ministry of Health; 2015.

[39] Federal Ministry of Health. Nigeria. National Strategic Health Development Plan (NSHDP) 2010 - 2015. Abuja, Nigeria: Federal Ministry of Health; 2010.

[40] Ekman B Liem NT Duc HA Axelson H Health Insurance Reform in Vietnam: A Review of Recent Developments and Future Challenges. Health Policy Plan. 2008;23:252-63. 10.1093/heapol/czn009 18424793

[41] Okpani AI Abimbola S Operationalizing universal health coverage in Nigeria through social health insurance. Niger Med J. 2015;56:305-10. 10.4103/0300-1652.170382 26778879 PMC4698843

[42] Uzochukwu BS Ughasoro M Etiaba E Okwuosa C Envuladu E Onwujekwe O Health care financing in Nigeria: Implications for achieving universal health coverage. Niger J Clin Pract. 2015;18:437-44. 10.4103/1119-3077.154196 25966712

[43] National Health Insurance Authority. National Health Insurance Scheme Technical Review. Accra, Ghana: National Health Insurance Authority; 2016.

[44] Mtei G Makawia S Masanja H Monitoring and Evaluating Progress towards Universal Health Coverage in Tanzania. PLoS Med. 2014;11:e1001698. 10.1371/journal.pmed.1001698 25244395 PMC4171093

[45] Nsouli S, Rached M, Funke N. The Speed of Adjustment and the Sequencing of Economic Reforms: Issues and Guidelines for Policymakers. IMF Working Paper No. 02/132. Washington, DC: International Monetary Fund; 2002.

[46] Kwon S Healthcare financing reform and the new single payer system in the Republic of Korea: Social solidarity or efficiency? Int Soc Secur Rev. 2003;56:75-94. 10.1111/1468-246X.00150

